# Role of pleural biopsy in etiological diagnosis of pleural effusion

**DOI:** 10.4103/0970-2113.71941

**Published:** 2010

**Authors:** Sudipta Pandit, Arunabha Datta Chaudhuri, Sourin Bhuniya Saikat Datta, Atin Dey, Pulakesh Bhanja

**Affiliations:** *Department of Chest Medicine, R.G. Kar Medical College, Kolkata, India*; 1 *Department of Medicine, North Bengal Medical College, Kolkata, India*

**Keywords:** Malignancy, pleural biopsy, pleural effusion, tuberculosis

## Abstract

**Background:**

Pleural effusion remains the most common manifestation of pleural pathology. Sometimes it is difficult to differentiate between tubercular and malignant pleural effusion in spite of routine biochemical and cytological examination of pleural fluid.

**Aims:**

This study aims to evaluate the role of pleural biopsy to determine the etiology of pleural effusion and to correlate it with the biochemical and cytological parameters of pleural fluid.

**Settings and Design:**

Seventy two consecutive patients of pleural effusion were selected from the out patient and indoor department of a tertiary hospital of Kolkata. It was a prospective and observational study conducted over a period of one year.

**Materials and Methods:**

Biochemical, cytological and microbiological evaluation of pleural fluid was done in all cases. Those with exudative pleural effusions underwent pleural biopsy by Abram’s needle. Subsequently, the etiology of effusion was determined.

**Results:**

Malignancy was the most common etiology, followed by tuberculosis. Pleural biopsy was done in 72 patients. Pleural tissue was obtained in 62 cases. Malignancy was diagnosed in 24, tuberculosis in 20 and non-specific inflammation in 18, on histopathological examination. Out of 20 histological proven tuberculosis cases adenosine de-aminase (ADA) was more than 70 u/l in 11 cases.

**Conclusions:**

In our study, malignancy is more common than tuberculosis, particularly in elderly. When thoracoscope is not available, pleural fluid cytology and pleural biopsy can give definite diagnosis. Pleural fluid ADA ≥ 70 u/l is almost diagnostic of tuberculosis, where pleural biopsy is not recommended.

## INTRODUCTION

Pleural biopsy is helpful to reach an etiological diagnosis of exudative pleural effusion, particularly when malignancy is suspected or when results of detailed pleural fluid study are inconclusive, especially in a set up where thoracoscope is not available.

Pleural effusion is the most common manifestation of disorders of the pleura. After routine and radiological investigations, the diagnostic work up of patients with clinically significant pleural effusion usually begins with a thoracentesis. Then on the basis of whether the fluid is transudate or exudate (according to Light’s Criteria), diagnostic insight is provided and further evaluation carried out.[1]

Whenever a diagnostic thoracentesis reveals an exudative effusion; more often than not, anti tubercular therapy is initiated, more so in a developing country like India.

This study was undertaken to determine the etiology of pleural effusion by various biochemical, cytological, histopathological and microbiological tests, and to perform pleural biopsy in those patients where indicated, in order to reach an etiological diagnosis of pleural effusion. Also, the etiology was corroborated with the levels of Adenosine deaminase (ADA) in pleural fluid and results analyzed.

## MATERIALS AND METHODS

This study was done in a tertiary hospital of Kolkata from May 2007 to April 2008. Seventy two consecutive cases of pleural effusion were evaluated clinically, radiologically and by thoracocentesis. Subsequently biochemical (protein, Lactate dehydrogenase, Adenosine deaminase), cytological (cell type, cell count and malignant cells) and microbiological (Ziehl-Nielsen stain) tests of pleural fluid were done to determine the etiology of effusion. Apart from the routine investigations, specific investigations were done according to the clinical setting. Then, after obtaining proper consent, pleural biopsy was done in all patients with exudative pleural effusion, using Abram’s pleural biopsy needle. Three biopsy specimens were obtained from each patient. They were sent for histopathological and microbiological (Z-N stain) examination for tuberculosis. Culture of pleural fluid and biopsy tissue for tubercle bacilli was not done due to lack of facility of rapid culture method (BACTEC). Conventional Lowenstein-Jensen (L-J) method was not done because it was time consuming. All the patients were closely followed up after pleural biopsy to observe for any complications and were managed accordingly.

Biochemical parameters of pleural fluid were determined using a selective discrete multi channel analyzer. Total protein concentration (g/dl) was measured by auto analyzer using Biuret method (Technicon RA 1000).[2], Lactate dehydrogenase (LDH) levels were measured by auto analyzer using modified IFCC (International federation of clinical chemistry) method (Technicon RA 1000)[2] and ADA level measured by auto analyzer using the colorimetric end point method described by Guisti and Galanti[3] (Microlab300, Merck, Netherland). Light’s criteria were used to distinguish exudates from trasudates.[1]

Malignant effusions were diagnosed if malignant cells were found in repeated cytological examinations (five times) or in histopathological examination of biopsy specimen.

Effusions were considered to be tubercular if plural fluid was positive for acid fast bacilli (AFB) by Z-N staining or pleural biopsy specimen revealed typical epitheloid granuloma consistent with tuberculosis (TB).[4] A serum ADA level of >40 u/l was taken as a cut-off value for diagnosis of tuberculosis.[1]

Parapneumonic effusions were diagnosed when an acute febrile illness was present along with purulent sputum, leucocytosis and pulmonary infiltrates in chest X-ray, in the absence of malignancy or other disease causing transudates.[5]

Lastly, the levels of ADA in pleural fluid and histopathology of pleural tissue were analyzed and the observations correlated. Study was been conducted after approval from ethical committee and written consents were been obtained from all patients before pleural biopsy.

## RESULTS

Out of 72 patients, the male: female ratio was 1.79:1, most cases being in the age group of more than sixty years. Among the histopathologically proven tubercular pleural effusion 71.4% cases presented with chest pain, breathlessness, dry cough and fever of which fever was the most common symptom observed. Fever and cough were the commonest symptom (69.2%) followed by breathlessness and chest pain (61.5%) in cases of malignant pleural effusion; 85.9% of cases had pleural fluid protein to serum protein ratio more than 0.5 and 64.1% of cases had pleural fluid LDH to serum LDH ratio more than 0.6. Cytology of pleural fluid revealed predominant lymphocytes in 52.6% cases and 39.7% cases revealed malignant cells in pleural fluid; 75 and 41% of clinically diagnosed tuberculosis and malignancy cases respectively had predominant lymphocytes in their pleural fluid. All 72 cases underwent pleural biopsy, among which pleural tissue was obtained successfully in 62 patients (86.1%) and among these 20 (27.7%) cases were tubercular and 24 (33.4%) cases were malignant histopathologically [Table 1]. Pleural tissue for acid fast stain was positive in 16 cases (22%). Histopathologically tubercular granuloma was found in all these cases. However acid fast stain of pleural fluid was negative in all the cases.

**Table 1:** Etiological diagnosis obtained by pleural biopsy and its correlation to pleural fluid Adenosine deaminase (ADA) level. (*n*=72)

Etiology	Total number of cases	Pleural biopsy histopathology		Adenosine deaminase U/L		
		No. of cases	Percentage (%)	<40	40 - 69	>70
Tuberculosis	22	20	27.7	-	9	11
Malignancy	38	24	33.4	20	4	-	
Chronic non -specific inflammation	18	18	25	4	14	-	
No pleural tissue	10	-	13.9	3	5	2

When the histopathological findings of tuberculosis were correlated with pleural fluid ADA levels, among all the histopathologically proved tuberculosis patients, nine patients had ADA level between 40-69 U/L. Thirteen patients had ADA level ≥ 70 U/L of which 11 had histopathologically proved tuberculosis and in the rest two patients, pleural biopsy tissue could not be obtained. None of the histopathologically proved tuberculosis cases had ADA level below 40 U/L. Among 27 patients with ADA level < 40 U/L, 20 were histopathologically proved to be malignant. On the other hand out of 24 cases of malignancy, 20 had ADA level below 40 U/L and four patients had ADA level in the range between 40-69 U/L [Table 1]. Chronic non-specific inflammation was the most common histopathological finding in the cases with pleural fluid ADA level between 40-69 U/L.

Out of 72 cases, a total of 38 were diagnosed as malignant, of which 24 were histopathologically proved by pleural biopsy and the remaining 14 were diagnosed by pleural fluid cytology. A total of 22 cases of pleural effusion were diagnosed to be of tubercular etiology of which 20 were histopathologically proved by pleural biopsy and remaining two cases by high ADA level (>70U/L).

## DISCUSSION

In our study the male: female ratio was 1.79:1. Similar observation was found in another study.[6] Majority of the patients in our study were aged 60 years or more although other studies presented a different picture.[7] This difference may be related to the fact that the study was done in a tertiary center and most of the pleural effusions in older age group are difficult to diagnose or remain undiagnosed in a primary or secondary health center and subsequently are sent to higher referral centre for further work up. In this study 71.4% of histopathologically proven tubercular pleural effusion cases presented with chest pain, dry cough, breathlessness and fever which correlates well with the study of Berger HW *et al*. (75%).[8] Chernow B *et al*. observed breathlessness as the commonest symptom (30%) in cases of malignant pleural effusion[9] but the present study reveals fever and cough to be the commonest symptoms (69.2%) followed by breathlessness and chest pain (61.5%). Yam LT *et al* have shown that predominant lymphocytes in pleural fluid are suggestive of either tuberculosis or malignancy in the majority of cases.[10] In the present study, 75% and 41% of diagnosed tuberculosis and malignancy patients respectively had predominant lymphocytes in their pleural fluid. Morrone N and Algranti E *et al*. performed pleural biopsy in 55 cases of pleural effusion and they found 43.6% cases were due to tuberculosis.[11] In a study from India, it was found that of the 50 patients of pleural effusion, 19(38%) were diagnosed as tuberculosis by pleural biopsy.[12] Mungal *et al*. performed his study on 55 cases of which malignancy was proved histopathologically in 47.3% cases.[13]

In another study, pleural biopsy established the etiology of pleural effusion as tuberculosis and malignancy in 31.1 and 22.4% of cases respectively.[14] In our study, pleural biopsy was done in 72 patients of whom 20 were tubercular (27.8%) and 24 were malignant histopathologically (33.4%). When compared with other studies, higher percentage of malignancy and lower percentage of tuberculosis cases found in our study may be due to the fact that ours being a tertiary care centre, more number of undiagnosed pleural effusion cases in older age group are referred to us from primary and secondary care centers for further work-up.

Acid fast stain of pleural biopsy was positive in 16 cases (22%), which correlates well with the study of Valdes L *et al*. (25.8%).[15] It appears that pleural fluid ADA level above 70 U/L is highly suggestive of tuberculous pleuritis where as pleural fluid ADA level below 40 U/L virtually rules out the diagnosis of tuberculosis.[1] This finding correlates well with our finding where thirteen patients had ADA level > 70 U/L and among them eleven were histopathologically proved tuberculosis; in nine histopathologically proved tuberculosis cases ADA levels were in the range 40-69 U/L. None of the malignant effusion or other diseases had ADA level > 70 U/L.

At the end, we can reach a definite diagnosis of tuberculosis and malignancy by pleural biopsy in 90.9% and 63.2% of cases respectively.

## CONCLUSION

In our country, at a tertiary care center, every pleural effusion is not due to tuberculosis and malignancy should always be excluded. Rather, incidence of malignancy may be more than tuberculosis, particularly in the elderly. When thoracoscope is not available, pleural fluid cytology and biopsy can give definite diagnosis in a significant number of cases of pleural effusion. Pleural fluid ADA level ≥ 70 U/L can be considered as diagnostic of tuberculosis and ADA level < 40 U/L virtually excludes diagnosis of tuberculosis.
